# Assessing Environmental Control Strategies in Cage-Free Egg Production Systems: Effect on Spatial Occupancy and Natural Behaviors

**DOI:** 10.3390/ani11010017

**Published:** 2020-12-24

**Authors:** Andrés F. Gonzalez-Mora, Araceli D. Larios, Alain N. Rousseau, Stéphane Godbout, Cédric Morin, Joahnn H. Palacios, Michèle Grenier, Sébastien Fournel

**Affiliations:** 1 Centre Eau Terre Environnement (ETE), Institut National de la Recherche Scientifique (INRS), 490 rue de la Couronne, Quebec, QC G1K 9A9, Canada; alain.rousseau@ete.inrs.ca; 2 Agri-Food Engineering Division, Research and Development Institute for the Agri-Environment (IRDA), 2700 rue Einstein, Quebec, QC GIP 3W8, Canada; araceli-dalila.larios-martinez.1@ulaval.ca (A.D.L.); stephane.godbout@irda.qc.ca (S.G.); cedric.morin@irda.qc.ca (C.M.); joahnn.palacios@irda.qc.ca (J.H.P.); michele.grenier@irda.qc.ca (M.G.); 3 Département des sols et de Génie Agroalimentaire, Faculté des Sciences de l’Agriculture et de l’Alimentation, Université Laval. 2425, rue de l’Agriculture, Quebec, QC, G1V 0A6, Canada; sebastien.fournel@fsaa.ulaval.ca; 4 Dirección Académica, Tecnológico Nacional de México, TecNM, Campus Perote, Km 2.5. Carretera Perote, México, Perote 91270, Mexico

**Keywords:** animal welfare, cage-free systems, laying hen behaviors, spatial occupancy, air quality

## Abstract

**Simple Summary:**

An increment pattern in the worldwide egg production, as well as in the farm’s capacity in laying hen housing systems, have been observed for the last fifty years. Also, animal welfare has become a subject of interest due to consumer awareness. These issues have introduced new challenges to respond to international markets’ demands while ensuring animal welfare and environmental footprint. Cage-free systems have been alternative systems to ensure the well-being of laying hens. Likewise, environmental control strategies have been implemented to improve air quality since airborne contaminants’ concentration can be high inside these systems. Thus, the analysis of the effect of these strategies on natural behavior and flock distribution is essential to assess a comprehensive analysis. Four strategies and a control were tested in an experimental cage-free system. Spatial occupancy and animal behaviors were tracked using video recordings. Neither the four environmental strategies nor the control applied in this experiment affected the natural behaviors of hens. However, changes in flock distribution and stress patterns were identified in the treatment with a reduction in litter allowance. This study provides evidence that it is possible to implement strategies to improve air quality without disrupting natural animal behaviors in cage-free systems.

**Abstract:**

Animal welfare concerns have been a challenging issue for producers and international marketing. In laying hen production, cage-free systems (CFS) have been identified as an alternative to ensure the laying hens’ well-being. Nevertheless, in CFS, important environmental issues have been reported, decreasing indoor air quality. Environmental control strategies (ECS) have been designed to enhance indoor air quality in CFSs. However, little information exists about the effect of these ECSs on natural animal behaviors. Four strategies and one control were tested in an experimental CFS, previously designed to track behavioral variables using video recordings over seven time-lapses of 1 hour per day. Spatial occupancy (SO) and laying hen behaviors (LHB) were registered. One statistical analysis was applied to evaluate the effect of ECS on SO and LHB using a multinomial response model. Results show lower chances to use litter area within the reduction of litter allowance treatment (T17) (*p* < 0.05). Neither the four ECSs nor the control implemented in this experiment affected the natural behaviors of the hens. However, stress patterns and high activity were reported in the T17 treatment. This study shows that it is possible to use these ECSs without disrupting laying hens’ natural behaviors.

## 1. Introduction

In the last fifty years, different laying hen production systems have been designed to improve animal welfare and respond to international market demands. Directives focusing on housing design and alternative methods to rearing laying hens were provided by the European Commission [1] to promote the egg industry’s minimum standards. Modifications included restricting the use of conventional cages, fostering experimental research, and encouraging political discussions on implementing enriched cages and cage-free systems (CFS) [2,3,4] 

CFS have been considered the best housing alternative for laying hens regarding animal welfare; allowing them to display a broader range of natural behaviors [5]. These systems provide litter areas, nest boxes, multi-level perches, feeders and drinkers, ramps, and novel objects, such as long sticks with color bands. Access to a litter surface allows hens to carry out natural and species-specific behaviors such as dust bathing, foraging, scratching, and pecking activities [6]. However, these activities can lead to environmental issues such as an increase in ammonia (NH_3_) emissions, airborne dust production, and undesirable indoor air quality for hens and workers [7,8].

High NH_3_ concentrations (>25 ppm) in CFS can induce respiratory disease to bird subjects to aerial viruses [9], and decrease in feed intake [10,11], as well as irritation of the trachea, ocular damage by corneal lessons [12], and increase in hen mortality [13]. Furthermore, hen behavior and spatial occupancy can be affected by NH_3_ concentrations where animals can reduce preening and foraging activities while in all likelihood preferring areas with low NH_3_ concentrations [14].

Airborne dust or particulate matter (PM) from litter disturbance can be transported as bio-aerosols carrying biological and organic substances (viruses, bacteria, fungal spores, gaseous pollutants, manure, and food residues), affecting the health of workers, hens, and people living close to confined laying hens housing systems [15,16,17]. Human and animal respiratory and heart illnesses have been linked to particle exposures less than 10 µm (<PM_10_). These particles can be inhaled, reaching the lower respiratory tract of humans (important residence time in the human body, might get into the bloodstream through the alveoli walls and create chronic diseases) [18,19]. Likewise, health concerns due to high dust levels in CFSs have been reported [20]. Hypotheses regarding the discomfort linked to the presence of airborne dust induced by laying-hen behavior have been made, however, there exists an evident lack of research activities to compare with other birds’ housing systems [21].

Factors favoring NH_3_ and PM emissions include litter properties; environmental conditions such as air humidity, temperature, and ventilation rate, free animal movements; as well as manure management systems [7,22,23]. To improve indoor air quality reducing dust and NH_3_, directly or indirectly, several environmental control strategies (ECS) have been suggested, namely: (i) incorporation of inert materials in aviary diet, e.g., biochar, zeolite or bentonite, to improve N retention in poultry manure [24,25]; (ii) litter amendment by adding natural zeolites, chemical compounds, i.e., aluminum sulfate or ferric compounds [26,27,28] or bedding materials such as white wood shavings or silage maize. [29]; (iii) sprinkling of neutral electrolyzed water [30]; (iv) negative and positive air-bulk ionization; (v) electrostatic precipitation; and (vi) oil spraying systems [31,32,33]. These ECSs have shown the potential to affect several aspects linked with dust and NH_3_ volatilization, namely: N retention in manure storage, poultry manure amendment, and particulate matter abatement. Nevertheless, to our knowledge, animal behavior assessment, more specifically on laying hen behaviors, has been the subject of few studies, and the focus has been on animal physiognomy alterations. Thus, this study aims to evaluate the effect of four strategies on spatial occupancy and laying hen behavior. The outcomes of this study are expected to contribute to the implementation of animal-friendly ECSs without negatively impacting their natural behavior and thereby the welfare in laying hen CFS.

## 2. Materials and Methods

### 2.1. Experimental Bench-Scale Rooms

The study took place in twelve independent bench-scale rooms (122 cm length, 119 cm wide). Each room was equipped with a variable-speed exhaust fan. The incoming air, drawn from outside, was preconditioned with an air conditioning unit and a heated electrical resistance unit to maintain an optimal temperature between 22 °C and 23 °C inside the rooms throughout the experimental period. The pre-conditioned air was distributed to all the rooms by using a series of ducts.

**Figure 1:**
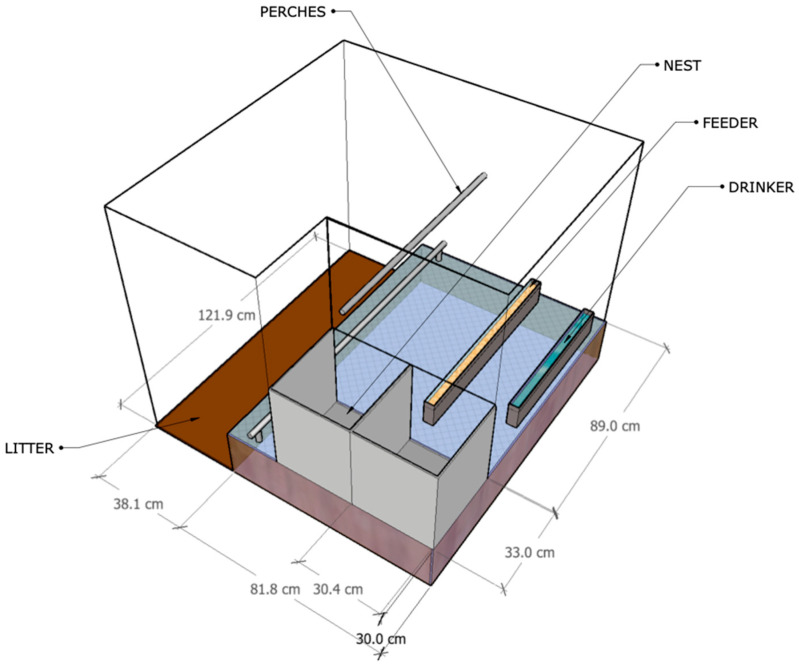
3D layout of an experimental room.

The rooms were laid out to meet the CFS housing requirements stipulated in the Code of practice for the care and handling of pullets and laying hens of the National Farm Animal Care Council of Canada (NFACC) [3]. Hens had free access to a wire floor space (122 cm long, 81 cm wide, and 30 cm high) and a litter space at floor level (122 cm length and 38 cm wide) (Figure 1). At the beginning of each experiment, the litter space was covered with a 5 cm thick wood shaving bedding. The wire floor was made with a square mesh wire cloth, and it was equipped with two nest boxes (30 cm by 33 cm) delimited by black plastic curtains and lined with a washable plastic mesh. The animal density was one hen per 1115 cm^2^. Two nipple drinkers and a linear feeder were installed in the wire floor space. The bird droppings collected beneath the wire floor were dried with forced air and removed once a week. A perforated 7.5 cm diameter duct blew air from a 10 cm blower (VTX-400, Atmosphere, Terrebonne, Quebec, Canada) fixed beneath the air inlet. Several holes (diameter: 5 mm, 160 mm apart), were positioned at a 45° angle. Then, based on airflow of 0.7 m^3^ h^−1^ hen^−1^, the blower was adjusted to obtain an air velocity of 2 m s^−1^. Two PVC-pipes (round profile) were installed over the wire floor area of each room (5 cm and 45 cm high), resulting in a linear space of 20 cm per hen.

### 2.2. Animal and Housing

Lohmann LSL-Lite laying-hens arrived at 19 weeks of age and were housed in the experimental rooms from February to June 2019 (n = 144, 12 hens per room). Trimming of the beak (at one-day-old) and vaccination requirements were done before their arrival at the experimental facility. Hens were individually weighted, placing the animals in a plastic recipient (8 L) with non-adjustable cover (to reduce stress) put on a digital platform scale previously tared. Then, the 144 hens were randomly selected to distribute them into the twelve experimental rooms. Four hens were randomly selected from each room and identified by painting a blue spot on their back. Further information about blue spot identification would be mentioned in Section 2.5.

An adaptation period of two weeks was provided at the beginning of the experiment. The two-week period allowed (i) the hens recognize their environment and (ii) the caretakers to carry out flash hen behavior visual observations, ensuring well-being conditions and animal adaptability.

Hens were fed with a commercial diet (Laying hen 18% VG, Agri-Marché, St-Isidore, Quebec, Canada). Feeders were filled once in the morning. Then, they were verified three times per day (by the caretakers) and refilled by the end of the day. Water was provided by a solenoid-activated valve connected to a data logger. Access to feed and water was ad libitum, and the amounts consumed were daily quantified for each room. The lighting intensity was 10 to 15 lux with equal lighting periods for all the rooms (Light Meter, Lux/FC, 840020C, Sper Scientific Ltd., Scottsdale, AZ, USA). An immediate on/off light cycle was set for each room.

### 2.3. Environmental Control Strategies (ECS)

Treatments included two combined ECSs previously selected by a panel of expert based on available literature. One treatment was proposed by the *Fédération de Producteurs d’Oeufs du Québec* (FPOQ, Quebec’s Federation of Egg Producers) to evaluate the possibility to decrease litter surface. Then, optimal parameters of the combined ECSs were selected following an experimental pre-test procedure focusing on economic and technical aspects (data not shown).

The four ECSs studied included: Decrease of litter surface area (T17); use of heated floor (HFOS), and litter amendment with biochar material (AOS) both combined with established oil sprinkling periods; as well as one single oil sprinkling treatment (OS). The experiment was divided into two consecutive experimental batches of eight weeks each (batch 1 and 2). A one-day time interval was used between batch 1 and 2 where the rooms were completely cleaned, ensuring any interference from the applied treatments in batch 1. The same laying-hens from batch 1 were housed for batch 2.

Table 1 provides a summary of the four treatments. One control (Ctrl) was settled during the experimentation with three repetitions (n = 3) for both batches 1 and 2, respectively. The control was a traditional aviary system with 33% of available litter surface. All treatments were applied to the litter space placed at floor level. It should be noted that AOS treatment was assessed in batch 1, while the OS treatment was evaluated only in batch 2. This experimental framework was selected to (i) evaluate the combination of applying acid adsorbent and sprinkling vegetable-oil, and (ii) the sole effect of sprinkled vegetable-oil, considering the number of experimental rooms and the replicate per treatment. The litter surface in all treatments was 33% of the total available floor area following the NFACC recommendations, except for treatment T17 where a reduced litter surface area was evaluated (17%). The wire floor area, nest boxes, perches, drinkers, and feeders followed the same specifications and dimensions for all the treatments as described in Section 2.1.

The HFOS treatment included the sprinkling of an acid emulsion made with vegetal oil and an organic acid solution. The application was carried out twice a week at a dosage of 585 mL m^−2^ of litter area. Sprinkling was carried out only on the litter surface. In this treatment, the underlying floor temperature was kept at 27 °C using an electrically heated floor system (EHF). The AOS treatment included the addition of an activated spruce-fir based biochar (Airex Energy Company, BiocharFX, Laval, QC, Canada), equivalent to 10% of the litter mass. The biochar particle size was ~1 mm, which was activated by acid protonation, after drying at 100 °C. The activation was made by immersion in a concentrated solution of sulfuric and nitric acid [34]. The OS treatment included the spraying of an acid emulsion twice a week, similar to the HFOS treatment, at a dosage of 585 mL m^−2^. In this case, the litter area was kept at room temperature. The mass of litter placed in each room was 900 g for T17 and 1800 g for the other treatments.

### 2.4. Spatial Occupancy and Laying Hen Ethology Analysis

Spatial occupancy and laying hen behavior were monitored for both 8-week experiments (batches 1 and 2). A video recording system (PoE Security Camera System 4CH, SMONET, Milpitas, CA 95035, USA) was set up inside the experimental rooms to record hens throughout the day. Four cameras were used for this study (POE Cameras) connected to a Network Video Recorder (NRV), saving 4TB HDD (Hard disk drivers) of data; that is, recording simultaneously four experimental rooms.

To observe spatial occupancy (SO), each bench-scale room was divided into six main areas: Nest (N), Litter (L), Perches (P), Feeder (F), Drinker (D), and Nest-feeder (NF). The N-region was obtained by subtracting the number of observed hens in the other areas from the total number of hens since it was not possible to count the number of birds inside the nest at each observation. The NF-region was established as the free space located between the feeders and the nest according to the room design (Figure 1).

Ten laying hen behaviors (LHB) were observed according to Blokhuis [35], Kristensen, Burgess, Demmers, and Wathes [14], and Weeks and Nicol [36]. Observed behaviors are described in Table 2. One additional behavior was added in the second batch to document any non-reported behavior (nBr) at the time of observation.

### 2.5. Video Recordings and Observation Analysis

**Figure 2:**
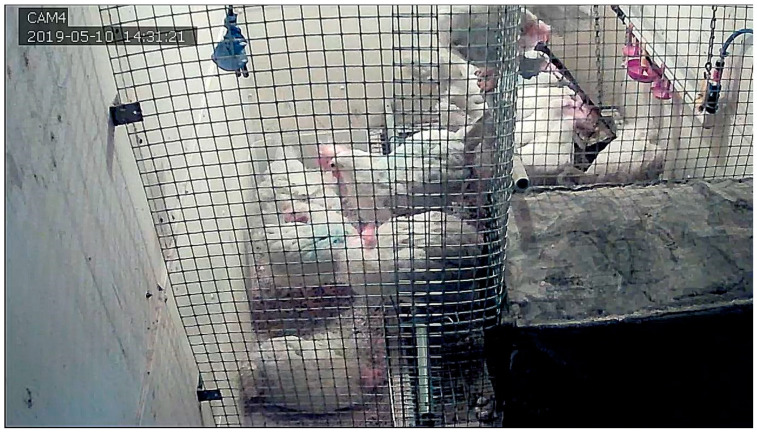
Video recording at an experimental room.

Two and three random days were selected for the video recording of each treatment for batch 1 and 2, respectively. One day of observations included seven 1-hour recordings covering most of the day, that is: 6 h–7 h/8 h–9 h/11 h–12 h/14 h–15 h/17 h–18 h/20 h–21 h/21 h–22 h. Each observation was made over a 1-min video at the half-way time of each 1-h recording by two trained observers. A total of 154 and 252 videos were analyzed for batches 1 and 2, respectively. The quantity of videos was not equal for both batches due to a lag time between the beginning of the batch 1 and the video recording system installation. Both SO and LHB were analyzed using the video recordings (Figure 2). For SO assessment, all the flock was considered for the score, while for LHB only four (4) laying hens, identified with the blue spot on the back, were observed.

The SO were assessed using the relative frequency of an event as proposed by Kozak et al. [40] where the number of hens per section over one day was divided by the total number of hens observed on the same day and multiplying by 100 to get a percentage (Equation (1)).

*SO*│*j* (%) = (*NoHi*/*THN*) × 100 (1)

where *NoHi* is the number of hens observed in the *i*-th section over one day, *THN* is the total number of hens observed on the same day, and *SO*│*j* is the relative frequency of spatial occupancy in the *j*-th day in the *i*-th section. Furthermore, LHB analyses were carried out by summarizing the number of hens doing a specific behavior divided by the number of experimental rooms undergoing the same treatment.

### 2.6. Statistical Analysis

The response variable of the SO analysis for each treatment was assumed to have a multinomial distribution. Therefore, a multinomial response model was applied using the PROC GLIMMIX procedure of SAS 9.4 (SAS Institute Inc., Cary, NC, USA) and the general logic link function. Treatment effect was tested with the F-test (*p* < 0.05). The response category having the highest relative frequency, “Perches”, was used as the baseline in the model. The odds that an animal under treatment A occupied i-space rather than reference space, i.e., perches, is the probability ratio Odds (Treat A).

Odds (Treat A) = Π*i,treat A*/Π*p,treat A*, (2)

where Π*i,treat A* and Π*p,treat A* are the relative frequencies of SO in the *i*-space and perches areas under treatment A. Treatment comparisons over spatial occupancy were performed using estimations of odds ratios (Equation (3))

OR (A,B) = Odds(Treat A)/Odds(Treat B)(3)

For the LHB, observed counts of each behavior were analyzed using a generalized linear model. The procedure PROC GLIMMIX of SAS version 9.4 (SAS Institute Inc., Cary, NC, USA, 2016) with logic link function was used to accommodate the assumption that counts have a Poisson distribution. The F-test for fixed effect was applied to assess the significance of the treatment effect on mean counts (*p* < 0.05). Mean counts per treatment for each behavior were estimated and its standard deviations (±) are presented, respectively.

## 3. Results

### 3.1. Spatial Occupancy (SO)

**Figure 3:**
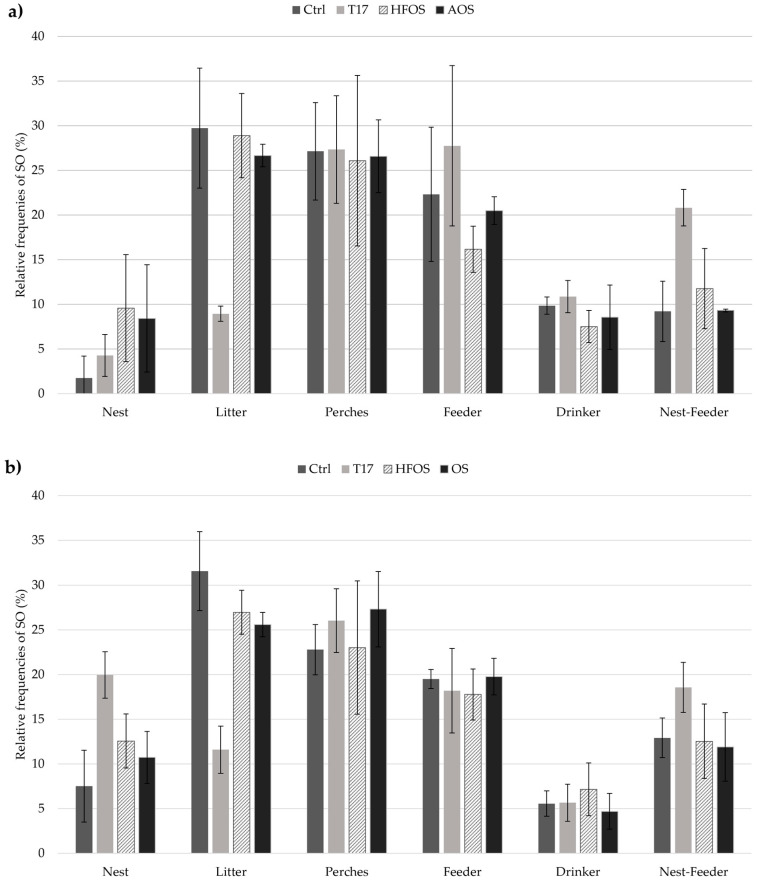
Relative frequencies of spatial occupancy (SO) with standard deviations from experimental cage-free system (**a**): Batch 1. (**b**): Batch 2. Ctrl = traditional aviary system. T17 = decrease of litter surface area. HFOS = use of heated floor with oil sprinkling periods. AOS = use of biochar material with oil sprinkling periods. OS = oil sprinkling periods.

The relative frequencies (%) of SO for the first and second batches are shown in Figure 3a,b. In the case of the first batch, values from the HFOS and AOS treatments have similar frequencies for all the six regions when compared to the control, except for nest area where larger values of 9.6% and 8.4% were calculated (Figure 3a). As mentioned before, it is noteworthy that frequency values in nest area were calculated by approximative subtraction since there was not any camera available inside the nest boxes. Low use of litter was observed in the T17 treatment, with a relative frequency of 8.9% ± 0.8%, compared to 26%–29% observed in the other treatments and the control. The odds of animal presence in the litter area were 3.45 times higher in control than that of the T17 treatment (*p* = 0.0001). It was observed that occupancy in feeders and nest-feeders area for the T17 treatment showed higher relative frequencies, with percentages of 27.8% ± 9.0% and 20.8% ± 2.0%, compare to 16%–22% and 9%–11% observed in the other treatments and the control, respectively. In fact, hens preferred the feeder area 1.69 times more in T17 when compared to the HFOS treatment (*p* = 0.04). Furthermore, the odds of occupancy in nest-feeders were 1.74 and 2.24 times higher in T17 treatment than that in the HFOS treatment (*p* = 0.05) and control (*p* = 0.0078).

For the second batch, similar relative frequencies were observed for the perches, feeders, and drinkers area for the three treatments and the control with average frequencies of 24.8% ± 2.2%, 18.8% ± 1.0%, and 5.8% ± 1.0%, respectively (Figure 3b). Moreover, a low-frequency value was found in litter area for the T17 treatment, with a percentage of 11.6% ± 2.6%, compared to 25%–31% observed in the other treatments and the control. The odds of animal frequency in litter area were 2.06 and 2.61 times higher for the HFOS (*p* = 0.0051) and OS (*p* = 0.0002) treatments than that of the T17 treatment. There was not any observed significant treatment effect in SO regarding random odds ratios between the HFOS and OS treatments, and the control.

### 3.2. Laying Hen Behavior (LHB)

**Figure 4:**
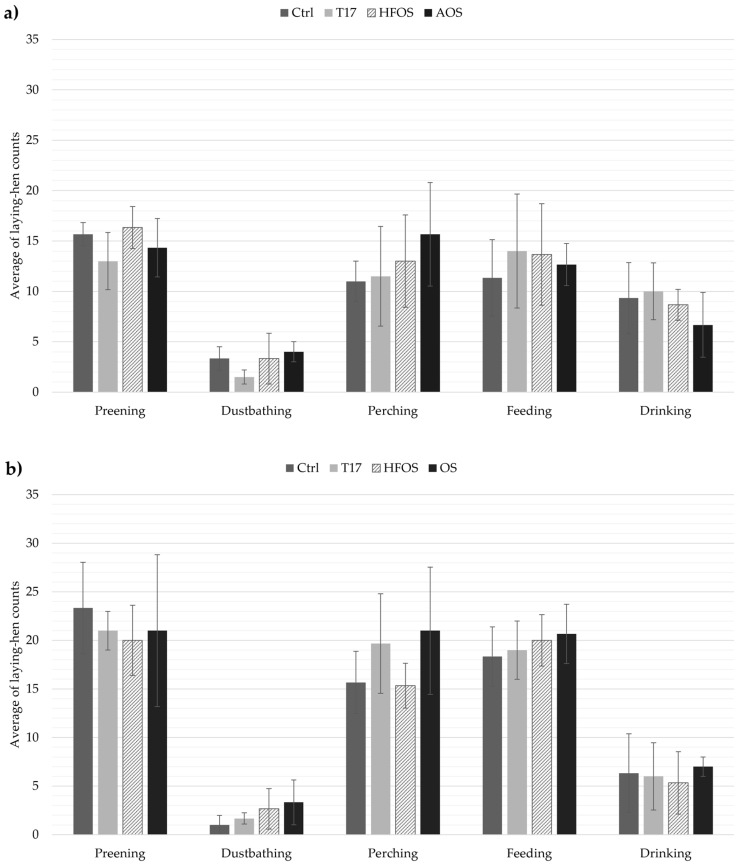
Average counts of laying hens displaying the five natural behaviors with standard deviations. (**a**): Batch 1; (**b**): Batch 2. Ctrl = traditional aviary system. T17 = decrease of litter surface area. HFOS = use of heated floor with oil sprinkling periods. AOS = use of biochar material with oil sprinkling periods. OS = oil sprinkling periods.

The average counts of laying hens performing a natural or species-specific behavior per day for batches 1 and 2 are shown in Figure 4a,b. Preening, perching, and feeding seem to be the most frequently observed behaviors reported in the experiment. Average values with standard deviations of 15 ± 2, 13 ± 2, and 13 ± 1 hens were observed doing these main activities for the three treatments and the control in batch 1. However, these behaviors had higher values in batch 2 (preening = 21 ± 1 hens, perching = 18 ± 3 hens, and feeding = 20 ± 1 hens). The difference between batches could be due to the number of observed days per room for the LHB analysis (Two random days for batch 1 vs. three random days for batch 2).

Species-specific behaviors such as dust bathing (DB) were reported for all three treatments and the control in both batches 1 and 2. These values were similar over the three treatments with average values and standard deviation of 3 ± 1 and 2 ± 1 hens observed for batches 1 and 2. Other animal behaviors, i.e., scratching, kneeling, ruffling feathers, body shaking, and drinking, did not show a particular trend. Furthermore, no significant effect of treatment was detected on any other behaviors following statistical analysis (Table 3).

## 4. Discussion

### 4.1. Space Occupancy (SO) Preference

Our results demonstrate that litter, perch, and feeder areas are the main locations where hens spend most of their time (Figure 3a,b). Litter and perch areas seem to be suitable to express the main natural behaviors that are preening, perching, and foraging [38]. Hens spent most of their time performing these behaviors.

The use of litter area was the lowest for the T17 treatment when compared to all other treatments and the control. There were 2 to 3 times more odds that hens would prefer litter in the other treatments. This means that reducing the litter surface area could lead to an unbalanced distribution of the flock within CFS. Furthermore, abnormal repetitive behaviors were identified while analyzing the video recordings for the T17 treatment, stress patterns as defined by Garner [41] were recorded. Thus, hens could be conditioned by the litter space available, promoting competition, and stress behavior inside the flock.

Displacement of animal behaviors could be observed if there were a motivational conflict. Taylor [42] defined this as normal behaviors taking place in an inappropriate situation, e.g., excess of feeding or preening. High activity of the flock and higher odds of feeder and nest-feeder area preference, observed in the T17 treatment, seems to be the result of motivational conflicts in the flock. However, it is recommended to assess more evidence to identify displacement behaviors for a litter reduction treatment (T17). It should be mentioned that anomalies within spatial distribution between nest and perch areas were observed as the light went out. In some cases, hens preferred rest in nest boxes than perched during the night. These distributions were attributed due to the shutdown lighting program which was configurated with immediate shutdowns.

### 4.2. Patterns in Animal Behaviors

Nesting activity in private places seems to be one of the preferred activities for laying hens, several studies have shown the preference for hens towards private spaces to lay eggs as a behavioral need [5,36,38]. Use of the nesting area was observed in this experiment with relative frequencies between 5% to 20%. In average, the use of nest area was similar compared to those relative proportions reported by Kristensen, Burgess, Demmers, and Wathes [14]. However, relative frequency of the nesting activity should be treated with prudence because these frequencies could be influenced by two aspects: (i) the short time lag used for observations, and (ii) the bird counts methodology in nesting area using an approximation by subtracting the number of observed hens in other areas from the total number of hens.

Preening, perching, and feeding were the most frequent behaviors observed in this experiment (Figure 4). These results are in agreement with Kristensen, Burgess, Demmers, and Wathes [14] who reported that about 14% of the time budget of laying hens is dedicated to preening. Also, Cordiner and Savory [43] showed an elevated proportion of time to perform perching activity during the day. Furthermore, perching behaviors are also related to preening or resting activities [35], which explains also high-frequency values for these behaviors.

In this study, natural and species-specific behaviors were observed for all treatments. There was not any significant effect linked to the four ECSs in any of the nine laying hen behaviors considered (Table 3). This was also reported by Engel et al. [44] who did not find any effect of litter allowance and nest-box availability on laying hen behaviors such as preening, scratching, etc., within modified cages. Other authors suggested a minimal effect on feeding, drinking, and resting behavior when space allowance is modified in furnished cages [45]. These results mean that to use ECSs for improving indoor air quality could be possible without affecting animal welfare, more specifically, with their natural animal behaviors.

## 5. Conclusions

Nowadays, animal behavior remains an important factor when assessing the quality of laying hen housing systems in light of animal welfare demands from markets and consumer’s needs. According to the literature, the application of ECSs inside these systems have proven the potential to improve air quality conditions for animals and workers in terms of airborne dust and gas concentrations. However, studies of the effect on laying hen behaviors are deemed necessary to better understand animal welfare.

This study provided an animal welfare analysis based on spatial occupancy and natural animal behaviors after the application of different ECSs. This article indicates that the natural behavior of laying hens exposed in a cage-free experimental system using different ECSs was not affected. However, the reduction of litter surface produced significant differences in the spatial distribution of the flock. Abnormal behaviors, as well as high activity in the flock have also indicated a possibly stressful environment for hens where litter surface was limited. Moreover, it is noteworthy that reduced litter allowance could be the limiting factor to trigger some behaviors performed in the litter area. Though there was not any significant effect observed over the laying hen behavior analysis, reduction of litter area is not recommended as animals could undergo stress patterns and unbalance spatial distribution. Besides, video recordings covering private places, i.e., nest boxes, could be useful to improve the analysis of spatial occupancy and animal behaviors. Also, gradual on/off lighting programs must be applied to reduce anomalies within spatial distribution in the flock while resting during the night. Additional (i.e., validation) research work should be conducted at an on-farm production scale with an online and integrated monitoring system to keep up with Precision Livestock Farming trends [46].

## Figures and Tables

**Table 1:** Environmental control strategies (ECS) applied at the experimental cage-free systems (CFS).

Batch	Abb. ^2^	Treatment	Description ^1^
1–2	T17	Decrease of litter surface area	17% of litter area, reduction of litter surface from 33% to 17%. n = 3 (rooms 1, 5 and 11).
1–2	HFOS	Heated floor + oil sprinkling	33% of litter area, installation of a heated floor fixed to 27ºC. Spraying an oily emulsion over litter (1.17 L/m^2^/week). n = 3 (rooms 2, 6 and 10).
1	AOS	Litter adsorbent + oil sprinkling	33% of litter area, addition of 10%-litter of acid adsorbent (Active biochar). Spraying an oily emulsion over litter (1.17 L/m^2^/week). n = 3 (rooms 3, 7 and 9).
2	OS	Oil sprinkling	33% of litter area. Spraying an oily emulsion over litter (1.17 L/m^2^/week). n = 2 (rooms 7 and 9).

^1^ Room design was based in a traditional aviary system [3]. ^2.^ Abb. = abbreviations. T17 = decrease of litter surface area, HFOS = use of heated floor with oil sprinkling periods, AOS = use of biochar material with oil sprinkling periods, OS = oil sprinkling periods.

**Table 2:** Laying hen behaviors observed inside all experimental CFS.

Behaviors	Description
Scratching	Bird scratching itself or scratching the litter with its feet. [37]
Kneeling	Includes events when the hen lies down over the litter, wire floor, or even on perches in a relaxing position.
Ruffling Feathers	The hen ruffles the feather without shaking its body. This activity could be observed in a standing or sitting position.
Body Shaking	Similar to RF but with an instant shaking movement.
Preening	The beak is in contact with the feathers. [35]
Dustbathing	Sitting position and arbitrary movements where the body, the feathers, the legs, and the beak could be in contact with the litter. [38]
Perching	A hen is stand up or sitting on a perch more than 2 s.
Feeding	Head in the feeder trough ingesting food. [39]
Drinking	Beak within the plane of the drinker. [39]
Supplementary behaviors	
Other	Other behaviors such as fluttering (wing flapping), sleeping (head through the feathers above the wing base), pecking or foraging, and stretching feathers.
Non-reported behavior (nBr)	A hen does not show any of the other behaviors. Stationary or motionless at the time of observation.

**Table 3:** F test for treatment effect on behavior counts in laying hens.

	Batch 1		Batch 2	
	F-Value	Pr < F	F-Value	Pr < F
Scratching	0.36	0.78	1.75	0.23
Kneeling	0.06	0.98	1.33	0.33
Ruffling Feathers	1.65	0.26	0.63	0.62
Body Shaking	0.94	0.47	0.20	0.89
Preening ^1^	0.35	0.79	0.28	0.84
Dustbathing ^1^	0.77	0.55	1.38	0.32
Perching ^1^	0.97	0.46	1.35	0.33
Feeding ^1^	0.30	0.82	0.17	0.92
Drinking ^1^	0.65	0.61	0.23	0.87

^1^ Behavior selected for depth analysis.

## Data Availability

The data presented in this study are available on request from the corresponding author with the previous authorization of Stéphane Godbout, IRDA researcher. The data are not publicly available due to their confidential nature, and they belong to the IRDA research group in charge of this study.
